# 

**Published:** 1887-07-30

**Authors:** 


					r
PRICE ONE PENNY.
?No. 44, Vol. II.
^(kt.
July 30th, 1887.
a ?J   .
8t?red at the General Post Office
as a Xewspaper.]
Per Annum, 6s. 6d.,post free.
Monthly Parts, 6d.; post free, 7?d.
Ditto per Ann., 7s. 6d.. post free.
ibihbmm ?
y ______
tfcg- mssaemml*-&m3satse*
~aSS '^Ca^ndl/orvr^\ //nrnrfnj.
an Institution, .jfamilg, antr $arocf)tal journal of
j&ogpttals. gswlums. & all agencies for Hie (ffarc of tfte jfrtcfe, Criticism & fletos

				

